# The African Vaccine-Preventable Diseases Network: a vaccine advocacy initiative

**DOI:** 10.4314/pamj.v8i1.71081

**Published:** 2011-03-14

**Authors:** Charles Shey Wiysonge, George E Armah, Shabir A Madhi, Fredrick Were, Sabrina Bakeera Kitaka, Chantal Akoua-Koffi, Gérard Gresenguet, Zipporah Gatheru, Patrick Wamae Maranga, Alassane Dicko, Adegoke Gbadegesin Falade, Angeline Yvette Crescence Boula, Stephanus Benjamin Kuit, Olumuyiwa O Odusanya, Papa Salif Sow, Nuruddin Lakhani, Evans Mwila Mpabalwani, Jean-Jacques Muyembe Tamfum, Gregory D Hussey

**Affiliations:** 1 School of Child and Adolescent Health and Institute of Infectious Disease and Molecular Medicine, University of Cape Town, Cape Town, South Africa; 2 Noguchi Memorial Institute for Medical Research, University of Ghana, Legon, Ghana; 3 Department of Science/National Research Foundation: Vaccine Preventable Diseases, University of the Witwatersrand, Johannesburg, Republic of South Africa; 4 Department of Paediatrics and Child Health, College of Health Sciences, University of Nairobi, Nairobi, Kenya; 5 Department of Paediatrics, Mulago Hospital and Makerere Medical School, Kampala, Uganda; 6 Institut Pasteur de Côte d’Ivoire, Abidjan, Côte d’Ivoire; 7 Faculty of Health Sciences, Bangui, Central African Republic; 8 Upper Hill Medical Centre, Nairobi, Kenya; 9 Network for Surveillance of Pneumococcal Diseases in the East Africa Region, Nairobi, Kenya; 10 Malaria Research and Training Centre, Faculty of Medicine Pharmacy and Dentistry, University of Bamako, Bamako, Mali; 11 College of Medicine, University of Ibadan, Ibadan and University College Hospital, Ibadan, Oyo State, Nigeria; 12 Mother and Child Centre, Chantal Biya Foundation, Yaounde, Cameroon; 13 Paediatrician, Bachbrecht, Windhoek, Namibia; 14 Department of Community Health & Primary Health Care, Lagos State University College of Medicine, Ikeja, Nigeria; 15 Département des maladies infectieuses, université Cheikh Anta Diop, Dakar, Sénégal; 16 Aga Khan Hospital, Dar es Salaam, Tanzania; 17 Department of Paediatrics and Child Health, University Teaching Hospital, Lusaka, Zambia; 18 National Institute for Biomedical Research, Kinshasa, Democratic Republic of Congo

**Keywords:** Vaccine preventable diseases, vaccine, network, Africa, awareness, child health

## Abstract

Achieving high and equitable childhood immunisation coverage in Africa will not only protect children from disability and premature death, it will also boost productivity, reduce poverty and support the economic growth of the continent. Thus, Africa needs innovative and sustainable vaccine advocacy initiatives. One such initiative is the African Vaccine-Preventable Diseases Network, formed in 2009. This association of immunisation practitioners, vaccinologists, paediatricians, and infectious disease experts provides a platform to advocate for the introduction of newly available vaccines (e.g. 10-valent and 13-valent pneumococcal conjugate and rotavirus vaccines) into the Expanded Programme on Immunisation (EPI) as well as increased and equitable coverage for established EPI vaccines.

## Background

It is already 11 years since the Millennium Development Goals were established and, with just four more years to go until the 2015 deadline for achievement, Africa is lagging behind the rest of the world in its commitment to reduce child mortality by two-thirds [1]. Vaccine-preventable diseases continue to be a major contributor to child mortality in the continent as a result of limited vaccine introduction and low immunisation coverage [2]. Inclusion of newly available vaccines against pneumococcal disease and rotavirus diarrhoea into the Expanded Programme on Immunisation (EPI) in African countries will reinvigorate efforts to achieve full and broad coverage of all available vaccines [2]. Pneumonia and diarrhoea remain leading causes of child deaths in Africa, accounting for over one-third of all paediatric deaths due to infectious diseases [1]. Newly available 10-valent and 13-valent pneumococcal conjugate vaccines are expected to provide broad protection against invasive pneumococcal disease in Africa, covering at least three-quarters of circulating serotypes [3]. In addition, two rotavirus vaccines have been studied in Africa and both vaccines show broad protection against disease-causing serotypes [4,5]. Despite a lower efficacy of these rotavirus vaccines in Africa compared to high-income countries, the number of preventable deaths is higher in Africa due to the exceptionally high levels of morbidity and mortality attributable to rotavirus diarrhoea in the continent [4,5]. There is thus a need for sustainable vaccine advocacy initiatives in Africa. One such initiative is the African Vaccine-Preventable Diseases (VPD) Network; formed on 30 October 2009 in Johannesburg, South Africa.

## The African Vaccine-Preventable Diseases Network

The African VPD Network comprises immunisation practitioners, vaccinologists, paediatricians, and infectious disease experts from across sub-Saharan Africa. The group endeavours to promote awareness of vaccine-preventable diseases as a prominent cause of morbidity and mortality in the African continent. The second meeting of the African VPD Network held in November 2010 in Cape Town, South Africa (on the heels of the Second World Pneumonia Day), established a framework for vaccine advocacy in Africa that will encourage the introduction of newly available vaccines and promote the uptake of existing and underutilised EPI vaccines.

Strategies for improved vaccine advocacy are needed in Africa, based on the knowledge that innovative and sustained activities can bring about change [6-9]. The group noted that increasing public awareness is critical for successful vaccine advocacy; as it is only by making vaccine-preventable diseases well known to the public that tackling them will become a national priority in each African country. Evidence-based information needs to reach all levels of society, from the general public to EPI decision-makers, dispelling any myths and misconceptions, and reminding stakeholders that vaccination is a safe and cost-effective health intervention.

## Conclusion

The positive effects of vaccination are far-reaching [10]. Immunising a nation’s children against diseases not only protects them from disability and premature death, it also boosts productivity, reduces poverty, and supports the economic growth of a country. The African VPD network is committed to improving access to vaccines via the development and publication of an advocacy action plan for Africa; identifying and opening dialogue with all immunisation stakeholders; and engaging with public figures outside the medical field, such as the media, who can champion the cause.

## Acknowledgement

The 1st and 2nd African VPD Network meetings were supported by an unrestricted educational grant from GlaxoSmithKline Biologicals. The funder had no role in the preparation or submission of this manuscript.

## Competing Interests

The authors have no competing interests.
